# Synthetic Food Colors and Neurobehavioral Hazards: The View from Environmental Health Research

**DOI:** 10.1289/ehp.1103827

**Published:** 2011-09-16

**Authors:** Bernard Weiss

**Affiliations:** Department of Environmental Medicine, University of Rochester, School of Medicine and Dentistry, Rochester, New York, USA

**Keywords:** behavior, effect size, food colors, hyperactivity, vulnerable populations

## Abstract

Background: The proposition that synthetic food colors can induce adverse behavioral effects in children was first enunciated in 1975 by Feingold [*Why Your Child Is Hyperactive*. New York:Random House (1975)], who asserted that elevated sensitivity to food additives underlies the signs of hyperactivity observed in some children. Although the evidence suggested that some unknown proportion of children did respond to synthetic food colors, the U.S. Food and Drug Administration (FDA) interpreted the evidence as inconclusive. A study published in 2007 [McCann et al. Food additives and hyperactive behaviour in 3-year-old and 8/9-year-old children in the community: a randomised, double-blinded, placebo-controlled trial. Lancet 370:1560–1567 (2007)] drew renewed attention to the hypothesis because of the study’s size and scope. It led the FDA to review the evidence, hold a public hearing, and seek the advice of its Food Advisory Committee. In preparation for the hearing, the FDA reviewed the available evidence and concluded that it did not warrant further agency action.

Objectives: In this commentary I examine the basis of the FDA’s position, the elements of the review that led to its decision and that of the Food Advisory Committee, and the reasons that this is an environmental health issue.

Discussion: The FDA review confined itself, in essence, to the clinical diagnosis of hyperactivity, as did the charge to the committee, rather than asking the broader environmental question of behavioral effects in the general population; it failed to recognize the significance of vulnerable subpopulations; and it misinterpreted the meaning of effect size as a criterion of risk. The FDA’s response would have benefited from adopting the viewpoints and perspectives common to environmental health research. At the same time, the food color debate offers a lesson to environmental health researchers; namely, too narrow a focus on a single outcome or criterion can be misleading.

In 1973, at a meeting of the American Medical Association, a retired pediatric allergist proposed a hypothesis that seemed ludicrous at the time. He claimed that at least some of the children labeled as hyperactive, or hyperkinetic, or afflicted with minimal brain dysfunction actually possessed an elevated sensitivity to certain elements of the diet. He followed with a book directed at the general public (Feingold 1975). He singled out food additives for special treatment. Feingold named artificial flavors and colors as primary culprits but also indicted some preservatives. Adopt a diet free of these offending ingredients, he advised, and many of the unsettling behavioral problems exhibited by these children will wane. Neither Feingold nor his critics defined hyperactivity in current terms of the American Psychiatric Association’s *Diagnostic and Statistical Manual of Mental Disorders* [DSM-IV (American Psychiatric Association 2000)]. The DSM-IV identifies three types of attention-deficit and/or hyperactivity disorder (AD/HD): predominantly inattentive (attention deficit disorder; ADD), predominantly hyperactive (attention-deficit/hyperactivity disorder; ADHD), and combined subtype (the most common). Boyle et al. (2011) give the proportion of U.S. children diagnosed with ADHD as 6.69%.

Feingold’s claims drew wide public attention and also provoked a series of studies directed at his hypotheses. Some tested elimination diets free of additives and other substances, such as salicylates, that Feingold linked to hyperactivity (e.g., Conners et al. 1976; Harley et al. 1978a, 1978b). Others focused on artificial colors because they represented only a small fraction of the additives in the food supply and could be manipulated more easily (e.g., Swanson and Kinsbourne 1980; Weiss et al. 1980; Williams et al. 1978). Most of these adopted the tactic of challenging children with one or a blend of food colors and a placebo. By the early 1980s, enough evidence about the Feingold hypothesis had accrued that even some of its most severe critics viewed it as plausible in some respects. For example, Stare et al. (1980) observed that “challenge experiments indicate that the symptoms of a small subgroup of all hyperactive children appear to be sensitive to the artificial food colors in their diet.”

After the early 1980s, interest in assessing behavioral reactions to food colors abated. During the intervening years, occasional studies both supporting and contradicting Feingold’s assertions about food colors made their way into the literature. From the point of view of the U.S. Food and Drug Administration (FDA), the positive studies taken together failed to constitute enough evidence to require regulatory action (FDA 2010). The data available up to 1982 (Weiss 1982) were considered not substantial enough to cause any shift in the FDA position. Debates about the behavioral toxicity of food colors continued but did not arouse singular interest.

The debate reignited with publication of a large study conducted by a group at the University of Southampton in the United Kingdom (McCann et al. 2007). It enrolled about 300 preschool and elementary school children who were challenged by a blend of food colors and sodium benzoate in a double-blind design employing a variety of behavioral measures. The study used two different mixtures, and the amounts chosen were based on estimates of intake by the British Food Standards Agency and probably are close to U.S. levels. Mix A included 20 mg artificial food colorings for 3-year-old children and 24.98 mg for 8- to 9-year old children. Mix B included 30 mg for the younger children and 62.4 mg for the older children. The doses for the 3-year-old children corresponded roughly to the amounts found in 112 g of candy.

The behavioral measures used by McCann et al. (2007), combined into a single score (as well as some components of the total score), demonstrated statistically significant adverse responses in both groups of children to the food color challenge. Although some of these measures are used in ADHD research and diagnosis, the Southampton study was aimed not at ADHD but at the more general question of behaviors evoked by food colors. Neither was the study aimed at the question of sensitivity to food colors in ADHD children; the subjects came from the general population of school children.

## Discussion

Because of the uniqueness and size of the study by McCann et al. (2007), it drew renewed attention to the food color debate, which was introduced to the environmental health community by an article in *Environmental Health Perspectives* (Barrett 2007), followed by a letter from Weiss (2008). Barrett (2007) solicited a response from a spokesperson for the FDA (Mike Herndon), who replied as follows:

However, we have no reason at this time to change our conclusions that the ingredients that were tested in this study that currently are permitted for food use in the United States are safe for the general population.

The article by McCann et al. (2007) elicited a petition to the FDA from the Center for Science in the Public Interest (CSPI), a public interest group that earlier had called for a ban on food colors (CSPI 2008). This petition, together with congressional interest and media publicity, led to an FDA decision to review the food color literature and to hold a public hearing before its established Food Advisory Committee. The hearing was held on 30–31 March 2011. After listening to testimony from FDA reviewers and the public, the committee concluded that the evidence was too inconclusive to link food colors to hyperactivity and too insufficient to recommend warning labels for products containing artificial food colors. (I testified before the committee that the available evidence indicated a connection between adverse behavioral responses and food color consumption.)

As described by the FDA Food Advisory Committee (2011a), the FDA framed the question put to the advisory committee primarily in the form, “Are food colors a cause of hyperactivity?” Only as a secondary question did the FDA ask if food colors might be a source of other kinds of adverse behavioral responses. Although the food color question was framed quite narrowly by the FDA, it is representative of many of the questions that confront the environmental health sciences. What kind of data—and how much data—does it take to render an outcome conclusive enough for action? The committee decision and the FDA’s current view (as quoted by Barrett 2007) signify a group of persistent questions pertaining both to environmental health science and to regulatory practices. In this commentary, I try to place the FDA committee decision in this broader context.

*Identifying the appropriate measures.* The FDA described the committee’s mission in these terms (FDA Food Advisory Committee 2011a):

The task before this Food Advisory Committee is to consider available relevant data on the possible association between consumption of synthetic color additives in food and hyperactivity in children, and to advise FDA as to what action, if any, is warranted to ensure consumer safety.

The charge did not explicitly conform to the DSM-IV definition of ADHD, which is multifaceted, so the charge was somewhat ambiguous.

Two review documents were contracted for and submitted to the Food Advisory Committee before the meeting: a background document, describing the FDA’s history of food color regulation (FDA Food Advisory Committee 2011a), and a literature review of publications about the connections between food colors and hyperactivity (FDA Food Advisory Committee 2011b). These documents provided the basis for the review presented to the committee by the FDA Office of Food Additive Safety.

In their review the FDA apparently decided to focus on Feingold’s 35-year-old hypothesis (Feingold 1975) rather than on the broader environmental issue of whether food colors may induce adverse behavioral responses. This is a broader issue because, as noted above, most U.S. children, not just those diagnosed with ADHD, consume synthetic food colors in their diet.

Moreover, few of the artificial food color challenge studies did so to test the hypothesis that food colors cause ADHD as defined by the DSM-IV. No one, of course, can specify any predominant cause of ADHD. It is clearly a multicausal disorder as well as one with notable variation in expression. The food color literature is aimed mostly at the short-term effects of challenges, not chronic disease. Although the questionnaires, rating scales, and performance assays prominent in ADHD research have proven useful in challenge studies, they do not encompass all the behaviors evoked by food colors. Swanson and Kinsbourne (1980) found that performance on a paired-associate learning task deteriorated after administration of a color mixture challenge. Goyette et al. (1978; see also Conners et al. 1976) identified 3 of the 16 children they assessed as responders by their performance on a visual tracking task. Even the FDA review observed that measures confined to ADHD symptoms may not reflect responses evoked by food colors. It noted the following in discussing a study by Rowe and Rowe (1994):

The behavioral effects elicited by the tartrazine challenges, however, involved irritability, fidgetiness and sleep problems which are not typically representative of hyperactivity related behaviors. Several other investigators also reported behavioral responses to color challenge that were not particularly characteristic of ADHD. (FDA Food Advisory Committee 2011b)

By narrowing the scope of the committee’s task to a judgment of whether artificial food colors are associated with ADHD, the FDA Food Advisory Committee (2011b) effectively eliminated a much more relevant and important question: Is there evidence that food colors are behaviorally toxic to the general population of children?

The large investment by the National Institute of Environmental Health Sciences (NIEHS) in bisphenol A research is, in many ways, a design for answering questions of similar scope. Bisphenol A is often labeled as “estrogenic.” Had the NIEHS bisphenol A initiative been restricted to this question (Spivey 2009), it might have limited its breadth only to questions bearing on the chemical’s alleged estrogenic properties. The NIEHS, however, recognized the scope of associations between bisphenol A exposure and health effects, including those such as obesity and externalizing behavior in young girls, that could not be linked firmly to estrogenicity, if at all. Analogously, if questions about the adverse health effects of airborne particulates had been restricted to lung function, the superficially obvious target organ, the association with cardiovascular function, its primary adverse effect, would have been overlooked.

One possible source of the FDA review’s misleading charge may be its limited view of brain–behavior relationships. In summarizing its findings, the FDA Food Advisory Committee (2011a) offered the following statement:

For certain susceptible children with attention deficit/hyperactivity disorder and other problem behaviors, however, the data suggest that their condition may be exacerbated by exposure to a number of substances in food, including, but not limited to, synthetic color additives. Findings from relevant clinical trials indicate that the effects on their behavior appear to be due to a unique intolerance to these substances and not to any inherent neurotoxic properties.

This statement surely does not mean to assert that the central nervous system is not the essential substrate for behavior or that behavior is a phenomenon independent of the brain. Its roots perhaps may be found in how toxicology was practiced in the past, when pathology—overt tissue damage—was far more important than function in assessing chemical safety.

*Identifying special populations.* The literature on behavioral toxicity of food additives is replete with observations by investigators—and by much of the applicable data—that not all children are sensitive to additives in general, or food colors in particular, at common dietary levels. Indeed, not even Feingold asserted that all hyperactive children were sensitive to food additives. In a convincing example of such findings, Rowe and Rowe (1994), in a double-blind controlled challenge study with tartrazine, identified a subgroup of 24 children within their sample of 54 that responded consistently on each occasion that they consumed a color rather than a placebo capsule. Moreover, these children displayed a clear dose–response function, with the higher doses eliciting higher scores on their 30-item behavior inventory, including five clusters of related behaviors: *a*) irritability/control, *b*) sleep disturbances, *c*) restlessness, *d*) aggression, and *e*) attention span.

The FDA review, however, seemed to insist that proving a connection between food color ingestion and adverse behavioral effects requires a uniformity of response in the sample under study that is virtually impossible to achieve in the diverse human population. For example:

Generally, the various reported findings across these 10 reviewed post-1982 portion of Group I trials, suggests that certain susceptible subgroups of problem behavior children with and without ADHD and, possibly, certain susceptible children from the general population without particular behavioral problems may exhibit a unique intolerance to artificial food colors resulting in typically small to moderate adverse behavioral changes which may not necessarily be characteristic of the ADHD syndromes. (FDA Food Advisory Committee 2011b).

Such a rejection of evidence stemming from data suggesting a subpopulation of children with enhanced sensitivity to food colors is perplexing. The FDA review implies that, because such a subpopulation may represent only a small proportion of children (hardly a proven proposition), it does not represent a significant health problem. Such a contention is inconsistent with the tenets of public health. Much of biomedical research, including environmental health research, is devoted to identifying and treating especially sensitive or vulnerable subpopulations. The underlying health goals of the Human Genome Project surely embraced that perspective. FDA drug warnings often are directed at special subpopulations. Finally, the FDA view on how this question pertains to food colors is an outlier among federal agencies. Note how the U.S. Environmental Protection Agency (2011) interpreted the Clean Air Act:

The National Ambient Air Quality Standards (NAAQS) are designed to protect the most vulnerable populations from outdoor air pollutants. Identifying these groups more precisely and understanding why they are more susceptible is of great importance to scientists and policy makers.

*Effect size.* In its critique of McCann et al. (2007), the FDA is somewhat dismissive of the results, at least as conveyed by this statement:

Whatever behavioral changes [in the Southampton study] may have occurred were apparently of rather low magnitude (effect size of 0.18). This would suggest that the type of treatment effects reported in this study, even though the investigators referred to increases in levels of “hyperactivity,” were not the disruptive excessive hyperactivity behaviors of ADHD but more likely the type of overactivity exhibited occasionally by the general population of preschool and school age children. (FDA Food Advisory Committee 2011b).

This is a puzzling statement because an important facet of an ADHD diagnosis is excessive or inappropriate activity. Also, the DSM-IV lists six kinds of hyperactivity, not just the variation described above. The term “occasionally,” in this context, is at least equally puzzling. Respiratory infections are also occasional events for most children. If a survey were to find, say, a significant rise in the incidence of such infections among a group of schoolchildren, questions would be asked and actions possibly taken. This question, in fact, is the theme of many reports in environmental health.

The more significant paradox about the passage above by the FDA Food Advisory Committee (2011b) is its view that an effect size of 0.18 [in the range of many of the published studies (see Schab and Trinh 2004; Stevens et al. 2011)] can be considered trivial. Effect size is often used to gauge the importance or strength of a finding; therefore, how it applies to McCann et al. (2007)—and its interpretation—is worth examining with a more familiar example.

**Figure 1:**
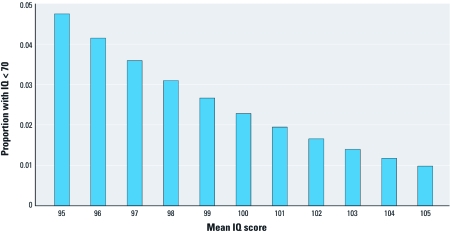
For an IQ distribution with a mean of 100 and SD of 15 (e.g., the Stanford-Binet), 2.3% of the population will have an IQ score < 70, a score that many school districts consider warranting remedial attention. If an environmental exposure shifts the mean IQ score by –3% (from 100 to 97), the proportion of the population with an IQ score < 70 will increase. Based on the current U.S. figure of 76 million children 0–17 years of age (Childstats.gov 2011), this represents an increase of 990,000 children in that category.

Consider Figure 1. For an IQ (intelligence quotient) distribution with a mean of 100 and and an SD of 15 (which describes a standardized IQ test such as the Stanford-Binet), 2.3% of the population will receive a score of < 70, a score that many school districts will view as warranting remedial attention. Now, define effect size, as used by McCann et al. (2007) and typically in the psychological literature, in terms of the standardized mean difference:

(mean 1 – mean 2) ÷ (pooled SD).

If an environmental exposure shifts the mean by 3%, equivalent to an effect size of 0.2, to a mean of 97, 3.6% of the population represented by the distribution will have a score < 70. Based on Census 2000 counts, the U.S. government (Childstats.gov 2011) estimates that there are 76 million children 0–17 years of age in the nation. Of these, 1.75 million would be presumed to have an IQ score of < 70, given a mean of 100. A shift of the mean IQ to 97 would indicate that 2.74 million children would have an IQ < 70 (an increase of 990,000 children). Most observers would not consider this to be a value of “rather low magnitude.”

**Figure 2:**
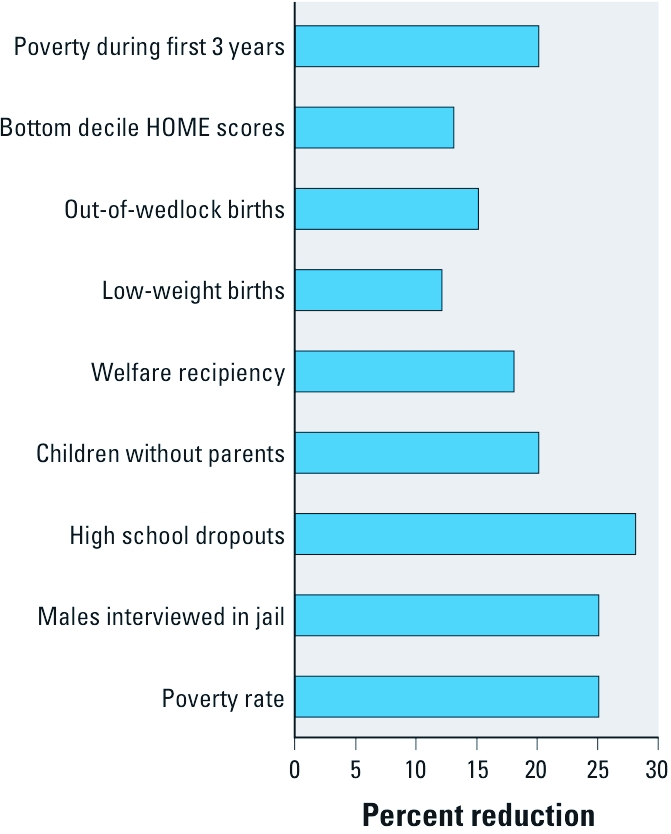
Depiction of calculations by Herrnstein and Murray (1994) of societal benefits achieved by a 3% rise in the population IQ.

Figure 2 presents another set of implications based on an effect size of 0.2, or a 3% shift in IQ. It depicts the calculations by Herrnstein and Murray (1994) of the broader social consequences of a population IQ increase of 3%, which was converted into the effects of a corresponding decrease in IQ by Weiss and Bellinger (2006). Although some of the presuppositions of these authors have aroused controversy, the relationships between IQ scores and lifetime earnings (e.g., Grosse et al. 2002), and how income influences the outcomes shown in Figure 2, lends credibility to the calculations.

One other aspect of effect size calculations that the FDA review failed to consider is how such values are influenced by population heterogeneity. In their Figure 2, Weiss and Bellinger (2006) showed how effect size calculations can be distorted if the sample population contains two subpopulations. Assume that the sample population consists of 70% nonresponders and 30% responders, and that the mean of the responders is shifted by 1 SD when presented with a challenge, such a food color. Under these conditions, it would require a total sample of 265 subjects to achieve an effect size of 1.0 as defined above. It is easy to see how true effects of a food color challenge to an unselected population can be missed if the sample size is small or if a minority of the sample consists of responders. Given such circumstances, it made sense for some investigators, such as Rowe and Rowe (1994), to screen subjects for responsiveness to an elimination diet before undertaking the tartrazine challenge portion of their study. It is analogous to the cancer bioassay strategy of using high doses to identify carcinogenic potential in reasonably small samples of rodents.

## Conclusions: The View from Environmental Health

The food color issue is emblematic of many questions in environmental health. What is the border between “inconclusive” and “conclusive” evidence? How is a susceptible population identified, and how large must it be for it to be seen as significant for public health? How broadly (or narrowly) should an outcome or criterion be defined and still remain relevant? From these standpoints, the FDA’s review, current position, charge to the Food Advisory Committee, and view of the issue’s future reflect a somewhat narrow vision. It is revealed in the conclusion by both the committee and the review (FDA FAC 2011c) that further research on the topic is necessary (committee members voted 93% yes and 7% no, when asked if more research was needed). This is hardly a statement to evoke disagreement, but consider the way such studies would have to be carried out. They would require institutional review board (IRB) approval. How would the investigator address the question of risk? How likely is it that an IRB would approve a study design in which the investigator states that, according to the published literature and the FDA, some children respond to a food color challenge with adverse behavioral effects? What, then, asks the IRB, is the purpose of the study? The difficulty in devising an argument for conducting a study that would satisfy most IRBs reveals the flaws in the FDA’s current position.

The next phase of the protocol would prove at least equally daunting. Parents would have to provide informed consent. As with the argument to the IRB, the parent would have to be made aware that food color challenges have been reported to induce adverse behavioral effects in some children. Would more than a small proportion of parents agree to have their child included?

Consider the cost of such a study. The Southampton study (McCann et al. 2007) enrolled about 300 children, about 150 of nursery-school age and about 150 in elementary school, and it used a blend of food colors. According to the principal investigator, the study cost about $1 million to complete. If the FDA demands that each of the certified colors be studied individually, the total cost would reach $7 million. Additional studies are unlikely ever to be performed on the scale of that performed by the Southampton investigators.

If the FDA had approached the food color question from an environmental health perspective, it would have enlisted a broad sample of scientists from a variety of relevant disciplines to examine the question. Its model would have been that exemplified by the April 2011 issue of *Environmental Health Perspectives*, which highlighted the health effects of airborne particulate matter. That issue of the journal contained articles addressing topics such as differential susceptibility in populations, the effects of different particulate matter components on mortality, cardiovascular effects, coronary heart disease, and respiratory health.

Had the FDA approached the food color question with the breadth of inquiry adopted in 1977, when a select committee reviewed the toxicity of food additives permitted under the GRAS (Generally Regarded as Safe) criteria (Siu et al. 1977), it would have arrived at a different conclusion. That committee, whose deliberations were supported by the FDA, looked beyond the simple question of “safety.” It enlisted public comment early in the process; made the penultimate draft of its report available to the public via the *Federal Register* and solicited comments; noted the importance of “psychotoxicology” in food safety evaluation; and emphasized the unique risks to neonates, a vulnerable group not considered in the FDA review but that is exposed to food colors.

If the FDA had given consideration to how the food color question might effectively be resolved, it might have adopted the vision described by NIEHS in the promise of Green Chemistry for Environmental Endocrine Disruptors, a meeting held in March 2011 in Sausalito, California (Schug 2011). In a parallel fashion, the FDA might have called for a “green chemistry” approach to synthetic food colors.

In defense of the FDA position, one might argue that the narrow scope of the review and committee charge simply were products of the CSPI petition (CSPI 2008). But the agency has had 35 years since the Feingold book (Feingold 1975) and 37 years since the GRAS report (Siu et al. 1977) to address the neurobehavioral toxicity of food colors. Perhaps a regulatory agency is not capable of being proactive. However, the British Food Standards Agency has advised parents to consider eliminating artificial food colors from the diet, and the European Union has called for eliminating six colors or listing on the label the warning that “[the color] may have an adverse effect on activity and attention in children” (Food Standards Agency 2011).
